# Lingual laser frenotomy in newborns with ankyloglossia: a prospective cohort study

**DOI:** 10.1186/s13052-022-01357-9

**Published:** 2022-09-05

**Authors:** Fabio Dell’Olio, Maria Elisabetta Baldassarre, Fabio Giovanni Russo, Federico Schettini, Rosaria Arianna Siciliani, Pietro Paolo Mezzapesa, Angela Tempesta, Nicola Laforgia, Gianfranco Favia, Luisa Limongelli

**Affiliations:** 1 Complex Unit of Odontostomatology, University of Bari Aldo Moro, Bari, Italy; 2 Department of Interdisciplinary Medicine, University of Bari Aldo Moro, Bari, Italy; 3 Neonatology and Neonatal Intensive Care Unit (NICU), University of Bari Aldo Moro, Bari, Italy

**Keywords:** Ankyloglossia, Breastfeeding, Newborns, Laser surgery, Laser frenotomy

## Abstract

**Background:**

The study aims to describe the lingual laser frenotomy perioperative protocol for newborns with ankyloglossia with or without breastfeeding difficulties developed by Odontostomatology and Neonatology and Neonatal Intensive Care Units of the Aldo Moro University of Bari.

**Methods:**

Authors carried out a prospective observational cohort study. Newborns with ankyloglossia (classified by using both Coryllos’ and Hazelbaker’s criteria) with or without difficult breastfeeding (according to Infant Breastfeeding Assessment Tool) underwent diode laser frenotomy (800 ± 10 nm; 5 W; continuous wave mode; contact technique; under topical anesthesia) and follow-up visits after seven and thirty days postoperatively. The authors analyzed as main outcomes the perioperative pain intensity measured by the C.R.I.E.S. scale, the occurrence of complications and quality of healing, the quality of breastfeeding, newborn’s postoperative weight gain, maternal nipple pain, and the presence of lesions as secondary outcomes.

**Results:**

Fifty-six newborns were included in the current study. Intraoperative mean pain intensity was 5.7 ± 0.5 points, resolved within thirty postoperative minutes. Observed complications were mild punctuating bleeding, carbonization of the irradiated site, and transitory restlessness. All wounds were completely healed within the thirtieth postoperative day. During follow-up, a significant breastfeeding improvement was evident with satisfactory newborns’ weight gain and a significant reduction of nipple pain and lesions (*p* < .05).

**Conclusion:**

Our lingual laser frenotomy protocol provided significant breastfeeding improvement in the mother-newborn dyads with low intraoperative pain and no significant complications.

## Background

Ankyloglossia, also known as “tongue-tie”, is a congenital anomaly meaning short lingual frenulum limiting mobility and impairing tongue function [1]; anyway, from 50 to 80% of newborns with ankyloglossia don’t show any problem. Some newborns who cannot hold nipples tightly with the tip and lateral margins of the tongue develop breastfeeding difficulties [2] and lingual frenotomy becomes an issue [3, 4]. Lingual frenotomy is the primary treatment for ankyloglossia with different reports about postoperative breastfeeding improvement [5–7]. Maternal nipple pain reduction, newborns’ weight gain, and improvement of milk intake are reported as other short-term effects of lingual frenotomy, but conclusive evidence is still lacking [7]. Since the first decade of 2000, several authors described the advantages of laser lingual frenotomy [8], but, according to the small number of studies, there are no definitive data about the possible superiority of the laser technique compared to the traditional surgical approach [9]. The current study describes the protocol of laser lingual frenotomy, developed by Odontostomatology and Neonatology and Neonatal Intensive Care (NICU) Units of the Aldo Moro University of Bari, in newborns with ankyloglossia with or without breastfeeding difficulties. The main outcomes are newborns’ perioperative pain intensity, the occurrence of complications, and quality of wound healing, whereas the postoperative quality of breastfeeding, newborns’ weight gain, maternal nipple pain, and lesions are considered secondary outcomes.

## Methods

A prospective observational cohort study was carried out in compliance with the principles of the World Medical Association Declaration of Helsinki on medical research protocols and ethics and the Aldo Moro University of Bari institutional review board (IRB) approved the study protocol (Study No. 4652, Prot. 1566/CE), developed by the Unit of Neonatology and NICU and the Unit of Odontostomatology. From September 2020 to September 2021, infants were included according to the following criteria: ankyloglossia according to Coryllos’ criteria with the indication for lingual frenotomy as determined by the Hazelbaker et al.’s Assessment Tool for Lingual Frenulum Function (ATLFF) [1], corrected gestational age > 40 weeks. Exclusion criteria were infants with age > 12 weeks [7] and newborns with craniofacial abnormalities, syndromes, or neurological diseases impairing their sucking ability [7, 10]. All newborns referred to the Unit of Neonatology because of ankyloglossia received an assessment of the lingual frenulum by a trained neonatologist by using Coryllos’ criteria (Table 1) for classification and Hazelbaker et al.’s ATLFF (Table 2) to determine the need for frenotomy. The latter is currently the validated [7] and complete assessment tool for tongue frenulum [1] and therefore the most reliable for determining a recommendation for frenotomy, showing high intra- and inter-observer agreement [11]. ATLFF is composed of a 7-item scale for tongue function assessment and a 5-item scale for tongue anatomy assessment; each item provides 0, 1, or 2 points, depending on the observed quality of function and anatomy; thus, function score ranges from 0 to 14 points and appearance score ranges from 0 to 10 points. According to ATLFF, there is a need for frenotomy if the function score is ≤ 10 points and the appearance score is ≤ 7 points [1]. Laser lingual frenotomy as treatment of ankyloglossia was performed after informed parental consent. Preoperative instructions were given to parents in terms of weighing the newborn the day before the procedure, and a preoperative fasting period (four hours in cases of breastfed infants and six hours for formula-fed infants) [10]. In the laser-safe operating room, the newborn was placed supine on the operating table and swaddled with the elbows held securely close to the body in a thermal blanket to prevent temperature loss. Heart rate and oxygen saturation with a pulse oximeter were constantly recorded during the procedure [10]. An assistant held and stabilized the head of the newborn while forcing the opening of the mouth by placing the index fingers between the patient’s upper and lower alveolar ridges. The oral surgeon used a micro-brush to apply a topical anesthetic (EMLA ®) on both the sides of the frenulum and its insertion on the tongue and the floor of the mouth. Laser frenotomy was started after five minutes from the topical anesthetic application with a diode laser (GaAlAs, wavelength 800 ± 10 nm – A2GLaser “Surgery 35”, A2G S.r.l., Italy) with a power output of 5 W, delivered by a flexible quartz optic fiber of caliber 320 μm in continuous wave mode with contact technique. The surgeon isolated the frenulum by using a straight clamp and the laser cut began from the free anterior margin of the frenulum and ended at its lower attach avoiding Wharton salivary ducts. After releasing the clamp, a diamond-shaped wound resulted, and the surgeon placed ice under the tongue for a minute and, in case of punctiform bleeding, applied a brief compression with sterile gauze for hemostasis. Soon after surgery, the mother provided postoperative breastfeeding and pulse-oximeter monitoring lasted for the following thirty minutes. Postoperative instructions were given to parents: to apply hyaluronic acid and amino-acids-based gel (Aminogam® gel) after each feed to protect the wound and stimulate the healing [12]; to administer acetaminophen in case of intense postoperative pain signs (e.g., inconsolable crying, restlessness); to report any complications, adverse events and/or complaints occurring during the thirty-day follow-up period. Before the discharge, two follow-up visits were scheduled (the first after seven days and the second after thirty days postoperatively) to evaluate newborns’ perioperative pain intensity, the occurrence of complications, and quality of wound healing as main outcomes, as well as the postoperative quality of breastfeeding, newborns’ weight gain, and maternal nipple pain and lesions as secondary outcomes. Pain intensity was measured by using the C.R.I.E.S. scale, a validated tool with high reliability [7], before surgery − as the baseline − , during surgery at the end of the surgery, and thirty minutes later. C.R.I.E.S. scale is represented in Table 3. The occurrence of complications was assessed both intraoperatively and postoperatively. Intraoperative complications considered were bleeding, injury of Wharton salivary ducts, and carbonization of the irradiated site [7]. Postoperative complications were bleeding and infection at the site of frenotomy (both wound and surrounding tissues) [7]. Quality of healing was assessed by using Healing Index by Landry et al. [13] on the seventh and thirtieth days after frenotomy (Table 4). The Healing Index score was treated as a quantitative outcome. Quality of breastfeeding was assessed during the first visit and on the thirtieth day after frenotomy, in partially or completely breastfed infants by administrating to mothers Infant Breastfeeding Assessment Tool (IBFAT) questionnaire [5], which is a validated 4 items-scale describing newborns’ behavior during breastfeeding; each item provides a score from 0 to 3 points, and total scores ≤ 8 identifies breastfeeding difficulties [1] (Table 5). Newborns’ weight (g) was measured on an empty stomach, without clothes and diapers by neonatologists in the Unit of Neonatology and NICU the day before frenotomy, seven and thirty days after laser frenotomy. Mothers’ pain experienced during breastfeeding and the occurrence of lesions were evaluated before the surgery and during follow-up visits. Nipple pain was evaluated with the Visual Analogical Scale (VAS). The two-tailed t-student test for paired groups was performed to compare quantitative measures recorded at different time points, by using IBM SPSS Statistics for Windows, Version 27.0, with an evidence level of *p* < 0.05.

**Table 1:** Coryllos’ classification of ankyloglossia

Type of Frenulum	Upper Insertion	Lower Insertion
1	On tongue tip	On alveolar ridge
2	On tongue tip	Just behind the alveolar ridge
3	In the middle of the tongue	In the middle of the floor of the mouth
4	In the middle of the tongue	In the tongue base

Coryllos’ classification of ankyloglossia recognizes four types of frenula based on the upper and lower insertions: type one has an attachment on the tip of the tongue and the other on the alveolar ridge; type two has the lower attachment on or just behind the alveolar ridge; type three has the frenulum extended from the middle of the tongue to the middle of the floor of mouth; type four is attached to the base of the tongue. The first and second types correspond to anterior ankyloglossia, whereas the third and fourth types (with functional impairment) correspond to posterior ankyloglossia

**Table 2:** Hazelbaker’s Assessment Tool for Lingual Frenulum Function

Function Items	Clinical Features	Points
Lateralization	Complete	2
	Body of tongue but not tongue tip	1
	None	0
Lift of tongue	Tip to mid-mouth	2
	Only edges to mid-mouth	1
	Tip stays at the alveolar ridge or rises only with jaw closure	0
Extension of tongue	Extends the tip over the lower lip	2
	Extends the tip over the lower gum only	1
	Neither of above	0
Spread of anterior tongue	Complete	2
	Moderate or partial	1
	Little or none	0
Cupping	Entire edge, firm cup	2
	The side edges only, moderate cup	1
	Poor or no cup	0
Peristalsis	Complete, anterior to posterior	2
	Partial: originating posterior to the tip	1
	None or reverse peristalsis	0
Snapback	None	2
	Periodic	1
	Frequent or with each suck	0
Appearance Items	Clinical Features	Points
Appearance of the tongue when lifted	Round or square	2
	A slight cleft in the tip apparent	1
	Heart-shaped	0
Elasticity of frenulum	Very elastic (excellent)	2
	Moderately elastic	1
	Little or no elasticity	0
Length of lingual frenulum when tongue lifted	> 1 cm or embedded in the tongue	2
	1 cm	1
	< 1 cm	0
Attachment of lingual frenulum to tongue	Posterior to tip	2
	At tip	1
	Notched tip	0
Attachment of lingual frenulum to tongue	To the floor of the mouth or well below the ridge	2
	Just below the ridge	1
	At the ridge	0

The Hazelbaker’s Assessment Tool for Lingual Frenulum Function (ATLFF) is composed of a 7-item scale for tongue function assessment and a 5-item scale for tongue anatomy assessment; each item provides 0, 1, or 2 points, depending on the observed quality of function and anatomy; thus, function score ranges from 0 to 14 points and appearance score ranges from 0 to 10 points. According to ATLFF, there is a need for frenotomy if the function score is ≤ 10 points and the appearance score is ≤ 7 points

*Abbreviation: ATLFF* Assessment Tool for Lingual Frenulum Function

**Table 3:** C.R.I.E.S. scale

Parameter	Definition	0 points	1 point	2 points
C	Crying	absent or low pitched	high pitched, easily consolable	high pitched, inconsolable
R	Requires oxygen to reach saturation over 95%	No	< 30% oxygen	≥ 30% oxygen
I	Increased vital signs	similar to baseline	up to 20% of baseline	over 20% of baseline
E	Facial expression	not grimaced	grimaced	grimaced with not cry vocalization grunt
S	Sleeplessness	constantly asleep	frequently awake	constantly awake

C.R.I.E.S. scale is composed of five items scoring from 0 to 2 points for each, thus measuring pain from 0 to 10 points. “C” means “crying”, which can be absent or low pitched (0 points), high pitched – as characteristic of pain – but easily consolable (1 point), or inconsolable (2 points). “R” means “requires oxygen to reach saturation over 95%” because of dyspnea induced by intense crying; the need for oxygen may be absent (0 points), < 30% (1 point), or ≥ 30% (2 points). “I” indicates an “increase in vital signs” – heart rate (HR) and blood pressure – compared to baseline; authors relied only on HR in the current study. Vital signs can be steady (0 points), increased < 20% than baseline (1 point), or increased ≥ 20% (2 points). “E” indicates “facial expression” of the newborn: the most frequently associated with pain is “grimace”; the latter is composed of brow lowering, eyes squeezed shut, deepening of the nasolabial furrow, open lips and/or mouth; grimace may be absent (0 points), alone (1 point), associated to not-cry vocalization grunt (2 points). “S” indicates the degree of “sleeplessness” distinguishing when the newborn is constantly asleep (0 points), frequently awake (1 point), or constantly awake (2 points)

*Abbreviation: C.R.I.E.S.* Cry—Requires Oxygen—Increased Vital Signs—Facial Expression – Sleeplessness, *HR* Heart Rate

**Table 4:** Healing Index by Landry et al.

Parameter	0 points	1 point
Color of tissues	Red > Pink	Pink > Red
Bleeding on palpation	Present	Absent
Granulation tissue	Present	Absent
Incision margins	Exposed connective tissue	Complete epithelization
Suppuration	Present	Absent

The Healing Index assesses the quality of healing ranging from 0 points, meaning poor healing, to 5 points, meaning excellent healing, by summing five items: color of tissues (red > pink = 0 points; pink > red = 1 point); bleeding on palpation (present = 0 points; absent = 1 point); granulation tissue (present = 0 points; absent = 1 point); incision margins (exposed connective tissue = 0 points; complete epithelization = 1 point); suppuration (present = 0 points; absent = 1 point)

**Table 5:** Infant Breastfeeding Assessment Tool

Items	3 points	2 points	1 point	0 point
To get the baby to feed, you:	Placed the baby on the breast as no effort was needed	Used mild stimulation such as unbundling, patting, or burping	Unbundled baby, sat baby back and forward, rubbed baby’s body or limbs vigorously at the beginning and during feeding	Could not be aroused
Rooting	Rooted effectively at once	Needed coaxing, prompting, or encouragement	Rooted poorly even with coaxing	Did not root
How long from placing baby on the breast to latch & suck?	0 – 3 min	3 – 10 min	Over 10 min	Did not feed
Sucking pattern	Sucked well throughout on one or both breasts	Sucked on & off but needed encouragement	Sucked poorly, weak sucking; sucking efforts for short periods	Did not suck

Infant Breastfeeding Assessment Tool (IBFAT) is a validated 4 items-questionnaire describing newborns’ behavior during breastfeeding on a scale from 0 to 12 points; each answer provides a score (0–3 points) proportional to the infant’s efficacy in breastfeeding. Scores ≤ 8 reveal difficult breastfeeding

*Abbreviations: IBFAT* Infant Breastfeeding Assessment Tool

## Results

**Fig. 1:**
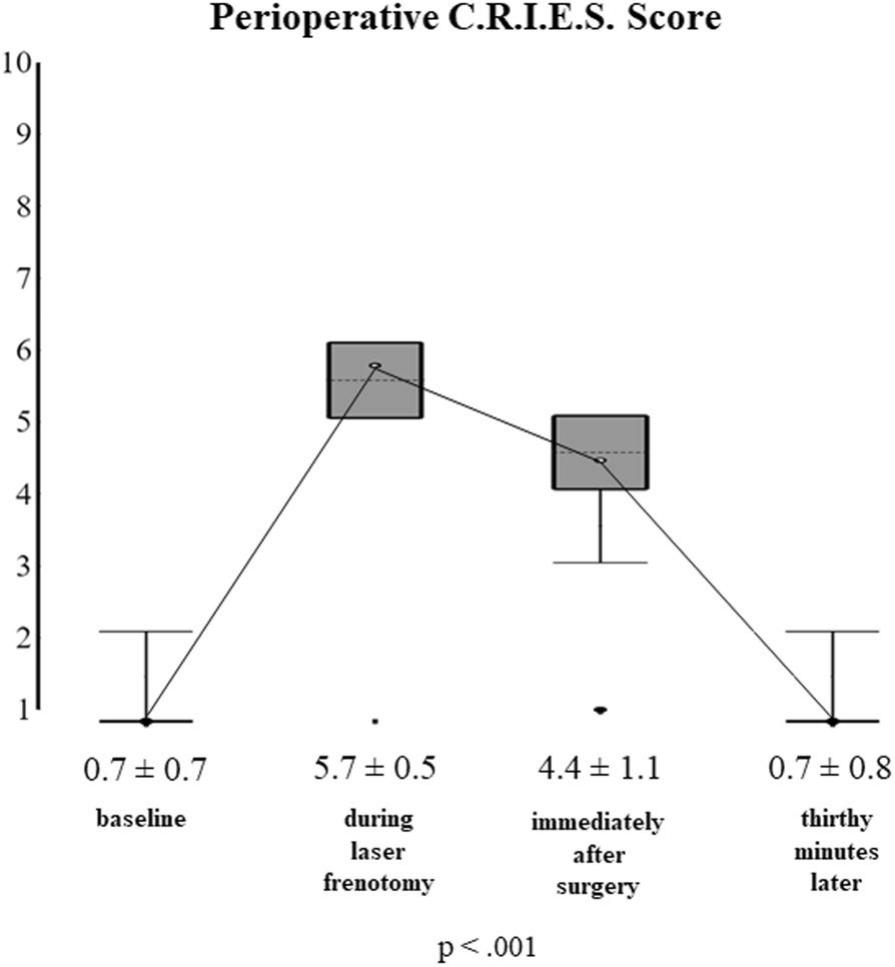
Perioperative Pain Intensity Curve. The current figure summarizes the variations in newborns’ pain intensity throughout the perioperative period (before surgery, during laser frenotomy, immediately after surgery, and thirty minutes after the end of surgery). The mean difference between each time-point and the following is statistically significant (*p* < .001). Abbreviations: *C.R.I.E.S.* Cry—Requires Oxygen—Increased Vital Signs—Facial Expression – Sleeplessness

**Fig. 2:**
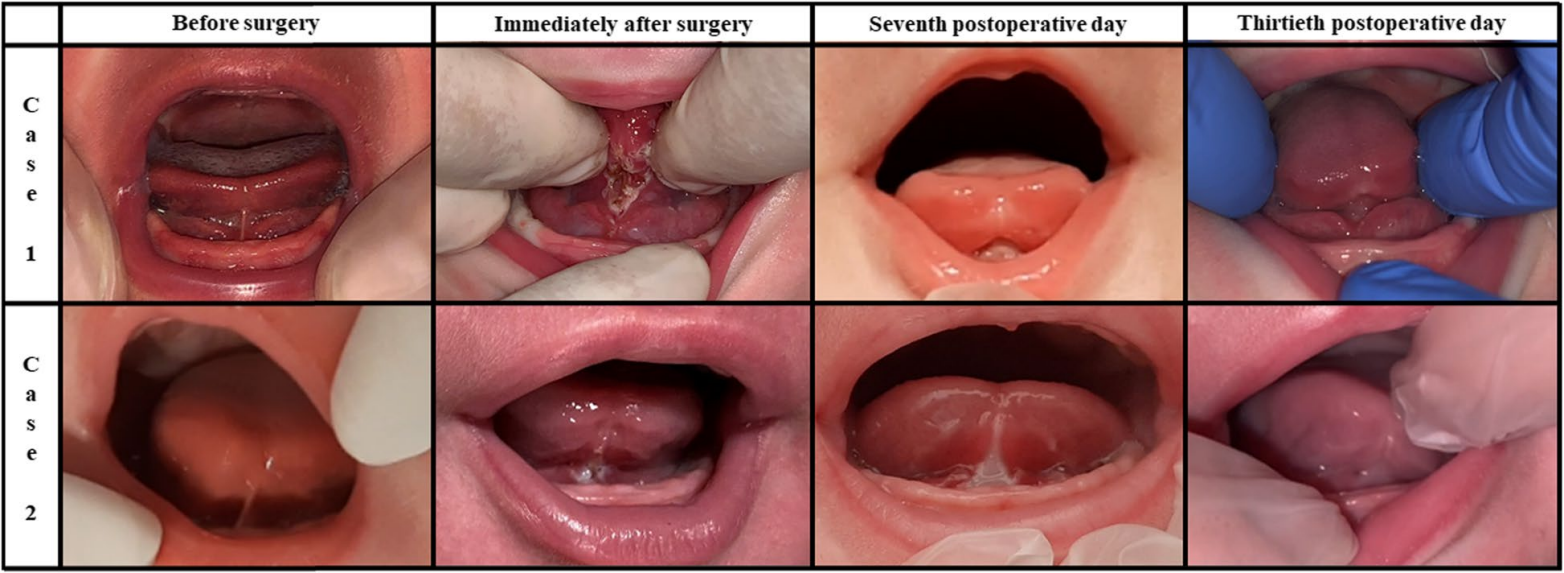
Lingual Laser Frenotomy - Wound Healing. The figure shows two examples among all included newborns; both cases comprehend preoperative, immediate postoperative, and follow-up pictures. Immediately after the laser frenotomy, all newborns showed diamond-shaped wounds covered by irradiated tissue. During the seventh postoperative day, the wounds were covered by a fibrin coat and surrounded by inflammatory erythema. During the thirtieth postoperative day, all newborns showed wounds covered by pink mucosa

**Fig. 3:**
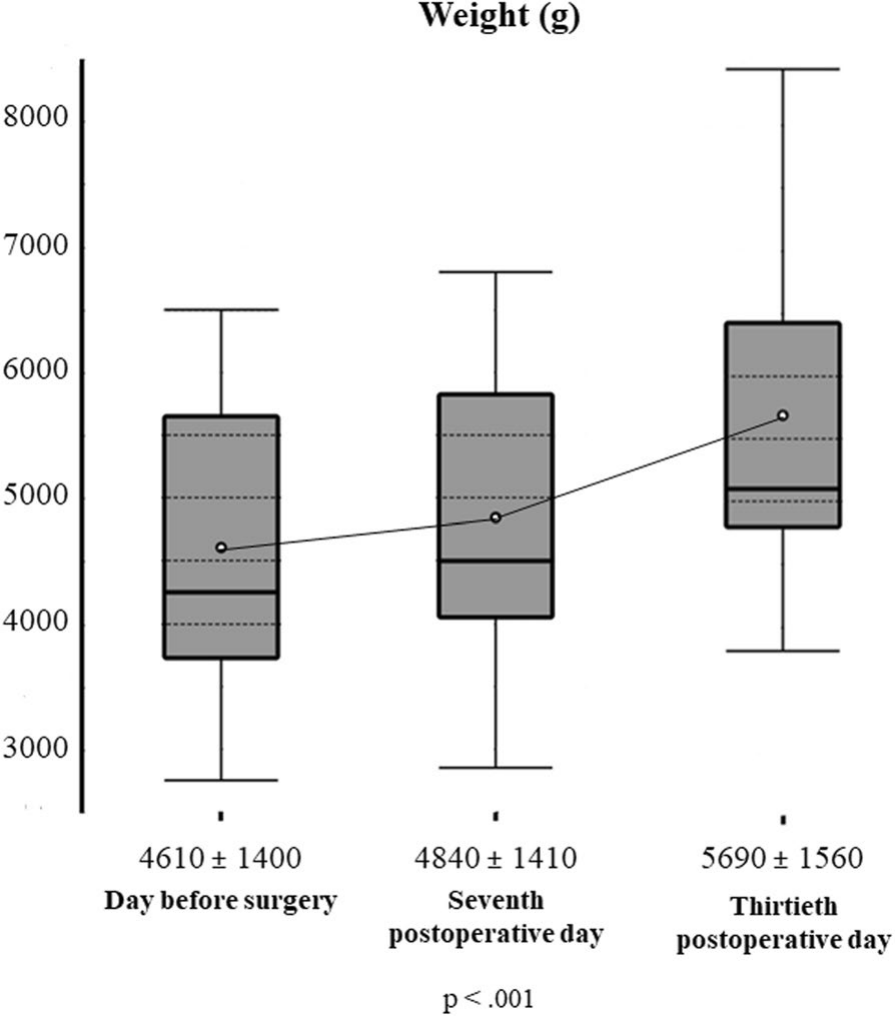
Treated Newborns’ Weight Curve. Newborns gained weight throughout the whole postoperative period: each box plot depicts data about weight (in g) recorded the day before surgery, during the seventh postoperative day, and during the thirtieth postoperative day. At each time point after surgery, the mean weight was significantly higher than the previous measurement, thus, the presence of surgical wounds did not harm the growth of the newborns. Abbreviations: *g* gram

**Fig. 4:**
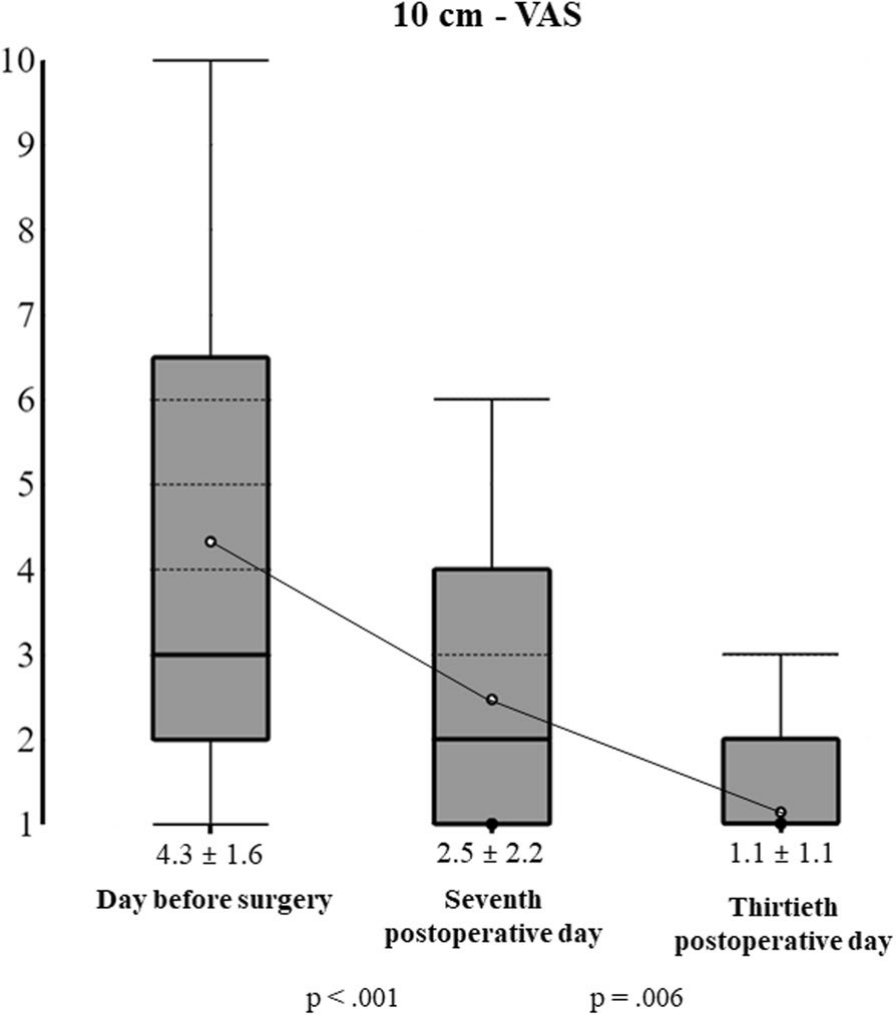
Nipple Pain (10 cm -VAS) Curve. Those boxplots depict how maternal nipple pain decreased throughout the postoperative period. The recorded mean VAS score decreased significantly both seven and thirty days after frenotomy. Furthermore, it is interesting to observe the maximum pain experienced by mothers: before surgery, some mothers reported 10 points; after one week, the maximum pain felt was measured at 6 points; after a month, the worst pain was 3 points. Abbreviations: *cm* centimeters, *VAS* Visual Analogical Scale

During the study period, 80 newborns were referred for ankyloglossia: 24 were excluded, 21 because no inclusion criteria were present, and 3 (with the inclusion criteria) because parents refused the laser procedure due to the absence of breastfeeding difficulties. Therefore, 56 newborns were included: 30 males (53.6%) and 26 females (46.4%). 34 (60.7%) were exclusively breastfed, and 22 (39.3%) received infant formula too. The mean age at frenotomy was 47.2 ± 20.2 days. According to Coryllos’ classification system, the cases of anterior ankyloglossia were 10 newborns (17.9%) with type 1 tongue-tie and 18 (32.1%) with type 2, whereas the cases of posterior ankyloglossia were 26 (46.4%) with type 3 tongue-tie and 2 (3.6%) with type 4. Their mean ATLFF function score was 7.8 ± 1.9 points whereas their mean appearance score was 4.8 ± 2.0 points. Their preoperative mean IBFAT score was 8.6 ± 1.9 points, and 24 (42.9%) had scores ≤ 8. None of the included patients showed lip-tie in addition to tongue-tie. All newborns completed the thirty-day follow-up. The analysis of pain intensity over time measured by using the C.R.I.E.S. scale is shown in Fig. 1. Before laser, the mean pain intensity was 0.7 ± 0.7 points as a baseline measure, based on the degree of sleeplessness: 26 (46.4%) were asleep, 21 (37.5%) partially awake and 9 (16.1%) awake, probably due to the hunger caused by the preoperative fasting period. During surgery, the mean pain intensity was 5.7 ± 0.5 points, the cry was high pitched and inconsolable in 47 newborns (83.9%), and easily consolable in 9 (16.1%). No oxygen desaturations (< 95%) were recorded during laser frenotomy. Hearth rate increased by more than 20% of baseline in 30 newborns (53.6%) and between 10 and 20% in 26 (46.44%). Facial expression was only grimaced in all newborns. After laser, the mean C.R.I.E.S. pain score was 4.4 ± 1.1 points. 54 newborns (96.4%) showed high-pitched but easily consolable cry, and only 2 did not cry (3.6%). 47 (83.9%) showed increased hearth rate < 20%, while 9 (16.1%) returned to the baseline levels. All newborns were awake, 37 completely (66.1%) and 19 inconstantly (33.9%). Thirty minutes after laser frenotomy, the mean C.R.I.E.S. score was 0.7 ± 0.8 points. None was still crying: 30 (53.6%) were constantly asleep, 15 (26.8%) frequently awake and 11 (19.6%) constantly awake. Pain intensity raised significantly during laser procedure (mean difference = 5 points; *p* < 0.001) and decreased significantly both immediately after frenotomy (mean difference = -1.3 points; *p* < 0.001) and thirty minutes later (mean difference = -3.73 points; *p* < 0.001). During frenotomy, the main complication (17 cases; 30.4%) was punctiform bleeding, while carbonization of the irradiated site occurred in 11 newborns (19.6%). No injuries to Wharton salivary ducts were recorded. At the seventh-day follow-up visit, one mother (1.8%) reported punctiform bleeding due to accidental trauma on the surgical wound that occurred during the first application of the protective gel. No infections at the site of frenotomy (both wound and surrounding tissues) were found. Refusal of the pacifier was reported in 39 cases (69.6%) and 15 (26.8%) were frequently awake as unexpected postoperative complications, anyway, none of the participating parents needed for administering acetaminophen to the infants for managing such signs of discomfort. During the thirtieth-day follow-up visit, newborns’ behavior was reported by all mothers to be not different from the preoperative period. Figure 2 shows the quality of healing after seven and thirty postoperative days. During the seventh-day follow-up visit, the surgical wound was covered by a fibrin coat with a variable red margin on peripheral mucosa with neither bleeding nor signs of suppuration, classified as discrete healing (Healing Index = 3 points). It is useful to report that many mothers noticed a white consistent “scab” on the site of frenotomy between the second or the third postoperative day that disappeared between the fifth and the sixth day. Such finding represents the progressive exfoliation of laser-treated tissue, part of the healing process. At thirty days after frenotomy, the quality of healing was excellent in all newborns (Healing Index = 5 points), because all surgical wounds were also covered by pink mucosa, without connective exposure and none of the newborns showed recurrence of ankyloglossia. At the thirty-day follow-up, all breastfed newborns showed improvement in the quality of breastfeeding: the mean IBFAT score was 11.7 ± 0.5 points, significantly higher than the preoperative record (8.6 ± 1.9 points; *p* = 0.001). Newborns’ weight variation is shown in Fig. 3. Preoperative mean weight was 4607 ± 1400 g; seven days after laser frenotomy was 4843 ± 1407 g and thirty days 5687 ± 1560 g, with significant weight gain in both visits (*p* < 0.001 and *p* = 0.001 respectively). Before surgery, 12 mothers (21.4%) had painful nipple lesions (e.g., fissures, inflammation, swelling); seven days after laser frenotomy, 10 (17.9%) still reported lesions, while only 2 (3.6%) at thirty days. Mean nipple pain was 4.3 ± 1.6 points before surgery, significantly reduced to 2.5 ± 2.2 points seven days after surgery (*p* < 0.001), and further reduced to 1.1 ± 1.1 points (*p* = 0.006) thirty days after surgery (Fig. 4).

## Discussion

The incidence of ankyloglossia ranges from 0.02% to 11.00% [2, 4, 7, 14–16] and several studies report male (3:1) prevalence [1, 2, 14–17]. Incomplete apoptosis of the anteromedial lingual prominence, over-fusion of the lateral prominences, and/or poor growth of anterior tongue length may be involved in the pathogenesis of ankyloglossia, with genetic factors probably involved in familial cases of ankyloglossia [1]. Ankyloglossia can also be found in many congenital syndromes such as Ehlers-Danlos, Beckwith-Wiedemann, Simosa, Simpson-Golabi-Behmel, Opitz, oral-facial digital syndromes, X-linked cleft palate, and in newborns from cocaine-addicted mothers [14, 16]. Anterior ankyloglossia causes significant limitation of tongue tip function, especially in protrusion, whereas posterior ankyloglossia impairs tongue function by its attachment to the middle of the tongue (the frenulum can be thickened or submucosal rather than short) [1]. Ankyloglossia can be asymptomatic and symptomatic because newborns can’t hold the nipple tightly with the tip and lateral margins of the tongue with breastfeeding difficulties [2]. Lingual frenotomy is indicated mostly because of difficult breastfeeding [3, 4], as poor sucking, prolonged and/or frequently interrupted feeding, nipple pain due to fissures, mastitis (even with bleeding), and obstruction of galactophorous ducts [11, 14, 18, 19]. Another indirect sign of difficult breastfeeding may be a newborn’s low weight gain as well as weight loss, with maternal anxiety and feelings of inadequacy [1, 4] and eventually depression [2, 8, 14–19]. The current literature lacks uniform diagnostic criteria and treatment indications, and it is stressed by many authors to perform a preoperative assessment before considering ankyloglossia as the real determinant of breastfeeding problems [2, 9, 18–20]. Among all classification systems and diagnostic criteria for ankyloglossia, ATLFF is frequently used but a correct breastfeeding assessment by reliable breastfeeding criteria such as IBFAT score or LATCH score − latch (L), audible swallowing (A), type of nipple (T), comfort (C) and hold (H), scoring from 0 to 2 points for each – is suggested [7]. According to the systematic review by O’Shea et al., the optimal age to perform lingual frenotomy is still unclear [7] but it is suggested to intervene before the development of atypical deglutition [16]. Neonatal lingual frenotomy is performed in neonatology or otolaryngology wards [7] as well as in outpatient settings [4, 16] (especially for thin lingual frenula [2]) and, according to a recent systematic review [15], is performed by surgeons, neonatologists, pediatricians, otorhinolaryngologists, obstetricians, lactation consultants, or pediatric dentists [16]. Many techniques for lingual frenotomy are currently used in newborns. The current study protocol used preoperative fasting to reduce the risk of regurgitation due to tongue stimulation during the laser application and to let the infants feed after surgery, which was important to stimulate the lingual function despite the presence of the wound; anyway, fasting remains uncommonly used. There is no definitive evidence about the best way to provide analgesia and/or anesthesia before lingual frenotomy in infants. Since injection of local anesthetic is not recommended in newborns [9], the laser lingual frenotomy is widely used with topical anesthetics [21]. Small amounts of topical anesthetics should be applied on mucosal surfaces to avoid possible toxicity (e.g., seizures, methemoglobinemia) [10], therefore their efficacy is unclear, and several authors do not recommend the use of such agents before frenotomy [9]. The same authors recommend the oral sucrose solution administration before frenotomy but acknowledge that its efficacy is debated, and a minimally effective dose is not known [9]. Therefore, the authors chose to apply the topical anesthetic to provide a superficial analgesic effect complying with the preoperative fasting protocol. The authors used EMLA ® as a topical anesthetic because it needs only two minutes of application to provide anesthesia on the tongue mucosa, the onset time is five minutes, and the application of this anesthetic cream is well-known in literature even if still off-label [10, 22]. Many authors have described laser lingual frenotomy as a relatively safe [17] and comfortable technique compared to traditional methods [14]. Economic costs [14, 23], risk of ocular inflammation due to exposition to light without protective glasses [18], rapid production of heat, need for external cooling [14], and the intrinsic risk of fire [18] are well-known disadvantages of the laser. American Academy of Pediatric Dentistry (AAPD) acknowledges laser as a surgical tool for newborns’ soft tissues [2] and diode, neodymium, CO_2_, and erbium are used, but diode lasers are widely used for the surgery of oral soft tissues, also for their relative costs [17, 23]. Tissue carbonization is the main complication related to laser frenotomy [14]. Lingual laser frenotomy requires deep study of the properties of the instrument and adequate training of the surgeon; furthermore, before surgery, parents must receive correct information and instructions to achieve the therapeutic goals [8, 14]. Actual evidence doesn’t support any specific standard postoperative protocol [9]. When applied for lingual frenotomy, diode lasers use the light of wavelength between 620 and 904 nm [23], 800–980 nm, or 1064 nm with contact or no-contact technique, by using power from 1.6 W to 3 W [8, 14, 16–19, 23, 24] conducted by a 320 µm quartz fiber [2]. During laser lingual frenotomy in newborns, the mucosa can be cooled by using wet gauze tamponing [17] other than ice tips, to prevent thermal damage [14]. Advantages of laser frenotomy comprehend brief-lasting surgery [2, 14–16, 18, 23], immediate hemostasis [2, 15, 18], low risk of bleeding [14], clear vision [15, 16], no need of suturing [2, 14, 16, 18, 23], less need of local anesthetics (in children) [2, 15], low postoperative edema and pain [14], less healing time [2, 14, 18], tissues sterilization – thus reducing the need of antibiotics and analgesics − [2, 14, 15], likely null risk of intraoperative bacteremia [14]. Furthermore, several reports show that the laser technique allows better wound healing than cold-blade surgery by exerting anti-inflammatory, bio-stimulant effects and reducing the colonization by myofibroblasts [14]. The systematic review by O’Shea et al. underlines the lack of perioperative pain assessment in different randomized controlled trials (RCTs) [7]. Modified Behavioral Pain Scale, Neonatal Infant Pain Scale, Neonatal Facial Coding System, or C.R.I.E.S. scale are all validated tools for measuring pain in newborns. C.R.I.E.S. scale shows 94% of inter-author concordance, and measures newborns’ pain by integrating both behavioral data (cry, facial expressions, sleeplessness) and vital parameters (HR and oxygen saturation) [7]; those were recorded in the current study by using a pulse oximeter perioperatively. We have shown that the laser lingual frenotomy performed with the preoperative application of lidocaine 2.5% + prilocaine 2.5% cream and postoperative ice caused low pain scores (measured by using a validated neonatal pain scale). Further randomized studies could measure the pain score of laser lingual frenotomy with and without co-interventions, and then understand the pain score caused by the surgery itself and the score reduction effect provided by the co-interventions. Newborns can be breastfed immediately after laser frenotomy with breast milk as an analgesic and antiseptic [4, 8, 18] or sucrose 24% solution can also be provided orally for analgesia [8, 18]. Mothers can be instructed to perform postoperative stretching exercises of the tongue to prevent the recurrence of ankyloglossia, such as lifting the tongue of the baby six times per day for three or four weeks [18]. After breastfeeding, the authors recommended the application of a hyaluronic-acid-based medical device (Aminogam® gel) because it protects the wounds and stimulates the healing of oral mucosa [12]. The use of hyaluronic-acid-based medical devices is safe and beneficial for alleviating oral symptoms in infants [25]. The real incidence of complications is biased by the lack of univocal diagnostic criteria and different classifications of ankyloglossia [15]. Posterior tongue-tie and cold blade-based techniques are reported to have more complications [5, 15] with possible damage around the lingual frenulum [18] due to the hypermobility of both the tongue and floor of the mouth [14]. Immobilization or good limitation of tongue movements is then a critical step during frenotomy [14]. The first postoperative assessment occurs immediately after frenotomy, to check for any bleeding [23] reported by different reviews, but of minimal significance [3, 7]. Within the first twenty-four postoperative hours, newborns may feel moderate pain that can be managed by administrating analgesics such as ibuprofen or acetaminophen [14, 19, 23, 24]. Several authors use acetaminophen as preventive analgesia four and eight hours after surgery [8]. During the first postoperative week, few infants were frequently awake in the current study, thus confirming the presence of persisting low-intensity pain. Other infants showed the refusal of the pacifier as the most common sign of discomfort due to the wound because it was likely a source of local trauma. This result is in line with the literature, which describes oral aversion (e.g., refusal of the pacifier) as a frequent complication after laser frenotomy [26]. The second postoperative assessment occurs a week after frenotomy, to check for possible postoperative complications [14, 16, 27], i.e. impairment of tongue movements [23], as well as to evaluate the healing process [18] that is completed within four weeks after laser [18, 23]. The frenotomy’s effect on weight gain remains to be clarified [5, 11, 19, 28, 29]. In the current study, weight gain was studied as a secondary outcome to confirm that laser frenotomy did not impair the infants’ growth, therefore didn’t account for covariates such as the infant age, birth weight, gestational age at birth, and type of feeding. The evidence about the immediate improvement in breastfeeding and maternal nipple pain reduction is not conclusive as well as for the duration of breastfeeding [5, 11, 19, 28, 29]. According to Messner et al. [9], informed consent for lingual frenotomy should include that the surgery could fail to improve breastfeeding and reduce nipple pain. Frenotomy is often requested by parents to prevent speech disorders, but it has been shown that there is no evidence of postoperative improvement in speech and, accordingly, frenotomy should not be performed [3, 4, 9]. Our study has some strengths: it has a prospective design with a follow-up rate of 100%; all newborns have been assessed by using multiple validated scales for diagnosis and classification of lingual frenulum anatomy, tongue function, and breastfeeding ability; it is the first non-randomized study to provide a detailed measurement of perioperative pain in newborns after lingual laser frenotomy.

## Conclusion

The current study supports laser frenotomy as an effective and safe treatment for newborns with ankyloglossia. Laser frenotomy caused low pain and very few complications both intra- and postoperatively with all wounds healed within thirty days with no recurrence of ankyloglossia. A significant improvement in breastfeeding and growth in newborns with a reduction of maternal nipple pain has also been shown.

## Data Availability

The datasets used and analyzed during the current study are available from the corresponding author on reasonable request.

## Abbreviations

NICU: Neonatal Intensive Care Unit
IRB: Institutional review board
ATLFF: Assessment Tool for Lingual Frenulum Function
C.R.I.E.S.: Cry—Requires Oxygen—Increased Vital Signs—Facial Expression – Sleeplessness
HR: Heart rate
IBFAT: Infant Breastfeeding Assessment Tool
VAS: Visual Analogical Scale
AAPD: American Academy of Pediatric Dentistry
LATCH: Latch—audible swallowing—type of nipple—comfort—hold
RCTs: Randomized controlled trials
BSES-SF: Breastfeeding Self-Efficacy Scale Short Form
NRS: Numeric Rating Scale
MPQ: McGill Pain Questionnaire
